# The signal that stimulates mammalian embryo development

**DOI:** 10.3389/fcell.2024.1474009

**Published:** 2024-09-17

**Authors:** Zoltan Machaty

**Affiliations:** Department of Animal Sciences Purdue University West Lafayette, West Lafayette, IN, United States

**Keywords:** oocyte, egg, sperm, fertilization, calcium, signal transduction, mammals

## Abstract

Embryo development is stimulated by calcium (Ca^2+^) signals that are generated in the egg cytoplasm by the fertilizing sperm. Eggs are formed via oogenesis. They go through a cell division known as meiosis, during which their diploid chromosome number is halved and new genetic combinations are created by crossing over. During formation the eggs also acquire cellular components that are necessary to produce the Ca^2+^ signal and also, to support development of the newly formed embryo. Ionized calcium is a universal second messenger used by cells in a plethora of biological processes and the eggs develop a “toolkit”, a set of molecules needed for signaling. Meiosis stops twice and these arrests are controlled by a complex interaction of regulatory proteins. The first meiotic arrest lasts until after puberty, when a luteinizing hormone surge stimulates meiotic resumption. The cell cycle proceeds to stop again in the middle of the second meiotic division, right before ovulation. The union of the female and male gametes takes place in the oviduct. Following gamete fusion, the sperm triggers the release of Ca^2+^ from the egg’s intracellular stores which in mammals is followed by repetitive Ca^2+^ spikes known as Ca^2+^ oscillations in the cytosol that last for several hours. Downstream sensor proteins help decoding the signal and stimulate other molecules whose actions are required for proper development including those that help to prevent the fusion of additional sperm cells to the egg and those that assist in the release from the second meiotic arrest, completion of meiosis and entering the first mitotic cell division. Here I review the major steps of egg formation, discuss the signaling toolkit that is essential to generate the Ca^2+^ signal and describe the steps of the signal transduction mechanism that activates the egg’s developmental program and turns it into an embryo.

## 1 Introduction

In sexual reproduction the union of the female and male gamete is a prerequisite for the generation of a new organism. The gametes must contain only half of the genetic material so that when they fuse together the diploid chromosome number is restored and the progeny will inherit genes from both parents. Also, during their formation the gametes must become capable of fertilization. This means that the spermatozoon must be able to reach the egg and activate its developmental program, while the egg must contain all the components of a signaling mechanism and all other molecules that are necessary to initiate embryo development. Development in the animal (and plant) kingdom is triggered by an increase in the cytosolic free calcium ion (Ca^2+^) concentration of the egg (Stricker, 1999). It is the sperm that stimulates the Ca^2+^ elevation and in mammalian species this Ca^2+^ signal takes the form of repetitive transients known as Ca^2+^ oscillations. In this review the formation of the mammalian egg will be described with special emphasis on the development of its signaling mechanism. The machinery that controls meiosis, the cell division of germ cells, will be explained briefly. The latest findings regarding the generation and maintenance of the Ca^2+^ signal will be presented and the major events that take place in response to the Ca^2+^ oscillations that culminate in the first mitosis of the newly formed embryo will be discussed. In this work the immature female gamete will be denoted as an oocyte. Once it completes meiotic maturation, extrudes the first polar body and arrests at the second meiotic metaphase it will be referred to as a mature oocyte or egg. For the purpose of this review the PubMed database was searched using keywords relevant to each section. Only those articles were included whose topic was related to the signaling process in the fertilized egg. I tried to provide a succinct story for each topic and make it readable and entertaining; if I left out anything or anybody, my apologies.

## 2 Oogenesis

### 2.1 Oocyte formation

Female gametes are highly specialized, terminally differentiated cells, whose single function is the propagation of life. They form in the fetal ovary through the process of oogenesis, from oogonia, which themselves develop from primordial germ cells. At one point during development oogonia enter meiosis, the cell division by which germ cells are produced. Meiosis encompasses one round of DNA replication followed by two rounds of chromosome segregation. The first round of meiosis is a reductional division, during which the homologous chromosomes (termed bivalents) are faithfully segregated so that only one copy of each chromosome remains in the oocyte and the other is discarded into the first polar body (Holt et al., 2013). Entry into meiosis involves a whole genome replication (S-phase) followed by a pairing of the homologous chromosomes. The homolog pairs then undergo double-strand DNA breakage during crossover, the “biological pinnacle of self-harm” (Sanders and Jones, 2018) and the subsequent repair of the breaks generates chromosomes made from both maternal and paternal components. In human germ cells about 2–3 crossovers take place between each pair of homologues (Alberts et al., 2002), resulting in a major shuffling of the genes. This increases genetic variation and thereby promotes evolutionary adaptation, which gives a huge advantage to sexual over asexual reproduction.

Meiosis is controlled primarily by the activity of a kinase, cyclin-dependent kinase 1 (Cdk1). In order to function as a kinase, Cdk1 requires the binding of cyclin B1, and together they form the maturation promoting factor (or M-phase promoting factor, MPF) with Cdk1 being the catalytic subunit and cyclin B1 the regulatory subunit. At this time MPF is inactive because Cdk1 is phosphorylated by an inhibitory enzyme, Wee1 and the cell is arrested at first prophase. Because of this meiotic arrest, the female germ cell, which is now a primary oocyte, spends the vast majority of its life at this state. Female mammals are born with a set of primary oocytes in their ovaries. The oocytes reside in primordial follicles, each ovary contains about 5,000 primordial follicles in mice (Bristol-Gould et al., 2006), 65,500 in sheep (Trounson et al., 1974), 66,500 in cattle (Erickson, 2010), 250,000 in pigs (Black and Erickson, 1968), and 500,000 in humans (Wallace and Kelsey, 2010). The germ cells at this stage have a large nucleus termed the germinal vesicle (GV), and they are arrested at the so-called GV stage, which is similar to the G2 phase of the mitotic cell cycle found in a somatic cell. The vast majority of these oocytes will undergo a process of cell death called atresia, the remainder will get periodically recruited into the growing follicle pool and will either be released from the follicle at ovulation or undergo atresia within non-ovulated atretic follicles (Holt et al., 2013).

### 2.2 Oocyte growth

At a certain stage during the reproductive cycle, oocyte growth is initiated. In the mouse, oocyte growth takes about 3 weeks and during this time the oocyte grows from 15 to 80 μm. In humans, growth takes approximately 6 months and the size of the oocyte increases from 35 to 120 μm. During growth, the oocyte accumulates all the transcripts essential for subsequent meiotic progression and fertilization (Sorensen and Wassarman, 1976). The cell cycle arrest is still maintained as the size of the oocyte, together with that of the follicle, increases. Limited Cdk1 and cyclin B1 availability seems to be a determining factor in the maintenance of this first meiotic arrest in many mammalian species (Fulka et al., 1988; Tatemoto and Horiuchi, 1995). However, as the oocyte grows, both proteins accumulate and acquire a greater capacity to associate with one another (de Vantéry et al., 1997). Once the oocyte reaches its final size and becomes meiotically competent, another regulatory system kicks in that helps to maintain the first meiotic arrest. This was demonstrated in rodents and cyclic adenosine 3′5′-monophosphate (cAMP) is the key player in the mechanism. cAMP is produced by the oocyte and its level is under the control of the surrounding granulosa cells (Hinckley et al., 2005). Granulosa cells produce another cyclic nucleotide, cGMP and after it is transported to the oocyte via gap junctions cGMP inhibits the hydrolysis (i.e., destruction) of cAMP (Zhang et al., 2010). cAMP keeps Cdk1 activity low by preventing its dephosphorylation (and thus activation), this blocks MPF activation and prevents entry into metaphase. The fully-grown oocyte also utilizes cyclin B1 degradation to keep Cdk1, and hence, MPF activity in check. Protein degradation is driven by the anaphase-promoting complex (APC), an E3 ubiquitin ligase that marks proteins for degradation by the 26S proteasome (Peters, 2006). APC requires the binding of a coactivator protein, either CDC20 or FZR1 (Holt et al., 2013). At this point it is FZR1 that controls the APC: oocytes lacking FZR1 undergo premature germinal vesicle breakdown (GVBD) in the presence of increasing cyclin B1 levels (Holt et al., 2011). FZR1-activated APC (APC^FZR1^) causes cyclin B1 degradation and in the absence of cyclin B1 Cdk1 cannot be activated, which leads to inactive MPF, and thus the first meiotic arrest is maintained until just before ovulation.

During growth, the primary oocyte acquires complex cytoplasmic organization and the cytoplasmic organelles become more abundant (Liu et al., 2006). In most species the centrioles disappear; the Balbiani body, a transient complex of diverse organelles forms around the nucleus; and the endoplasmic reticulum (ER) becomes more cortical. The number of ribosomes multiply, the Golgi apparatus enlarges and begins to export glycoproteins to form the zona pellucida and the cortical granules. The number of mitochondria, the organelle responsible for ATP production, also increases significantly. The mitochondrial DNA copy number, an indicator of the number of mitochondria in the cell, is 1,000 times higher in mature oocytes compared to primordial germ cells (Satouh and Sato, 2023). Morphology of the mitochondria also changes dramatically. At the time of oocyte formation, the diameter of the mitochondria in human oocytes is 1–1.5 μm with many cristae, whereas in fully grown oocytes the diameter is 0.4–0.6 μm with fewer cristae (Sathananthan and Trounson, 2000; Motta et al., 2000). All these events take place during the growth phase of oocyte development, which is extremely rapid and intense: mouse oocytes for example show a ∼300-fold increase in volume accompanied with a ∼300-fold rise in RNA content and a ∼38-fold elevation in the rate of protein synthesis (Wassarman and Albertini, 1994). Eventually, a gigantic germ cell is formed that is capable of becoming a viable embryo after fertilization.

### 2.3 Oocyte maturation

The final stage of oogenesis is oocyte maturation (or meiotic maturation). During maturation, the fully-grown oocyte acquires the ability to respond to the fertilizing sperm and produce the signal that initiates embryo development. The process involves two distinct yet interconnected events, nuclear and cytoplasmic maturation (Loutradis et al., 2006; Ferreira et al., 2009). Nuclear maturation encompasses germinal vesicle breakdown, chromosomal condensation and segregation, and extrusion of the first polar body, and as a result the diploid germ cell becomes haploid. Cytoplasmic maturation is less well defined but involves organelle reorganization, an increase in the content of intracellular Ca^2+^ stores and the storage of mRNAs and proteins that are required for the maturing oocyte and the early embryo prior to the activation of the embryonic genome (Yu et al., 2010).

The process is initiated by a luteinizing hormone (LH) surge from the pituitary gland that triggers the resumption of meiosis in the oocyte. The LH surge causes a decrease in NPR2 activity, the guanylyl cyclase that is responsible to produce cGMP in the cumulus cells. It also terminates gap junctions so the intercellular communication between the oocyte and the cumulus cells is lost. This results in reduced cGMP, and hence, low cAMP levels in the oocyte (Norris et al., 2008; Norris et al., 2009). Cdk1 is dephosphorylated (and thus activated) in the presence of low cAMP levels by the phosphatase called CDC25B (Lincoln et al., 2002); together this leads to high MPF levels that drive the cell cycle into metaphase I.

The increase in Cdk1 (and hence MPF) activity causes the dissolution of the nuclear lamins and subsequent germinal vesicle breakdown (GVBD; a G2/M transition), chromatin condensation and the formation of the first meiotic spindle (Sanders and Jones, 2018). In somatic cells, spindle formation is directed by the centrioles that promote the assembly of microtubules to form the bipolar spindle; oocytes lack centrioles and instead generate microtubule organizing centers (MTOCs) *de novo* that will guide spindle formation (Schuh and Ellenberg, 2007). The spindle apparatus harnesses each chromosome of the pair at random. The kinetochores of sister chromatids of each chromosome align side-by-side and orient towards the same pole, thus each homologue is separated from its partner and moved to opposite poles. This leads to a reductional cell division, i.e., the daughter cells end up with only half the number of chromosomes. The segregation of the chromosomes is controlled by the anaphase-promoting complex (APC), which is regulated, among other things, by the spindle assembly checkpoint (SAC). This mechanism prevents progress to anaphase until bivalents are properly captured on the spindle and has an important role in maintaining a high rate of faithful chromosome segregation in MI. The homologous chromosomes are held together by multisubunit protein rings called cohesins along the chromosome arms, these must be inactivated before chromosome segregation can proceed (Klein et al., 1999; Watanabe and Nurse, 1999). Inactivation is done by separase, an enzyme that is normally inhibited by securin. Once all the bivalents are correctly aligned and attached, SAC proteins allow for APC activation. Exit from metaphase also requires low MPF activity, which is achieved by cyclin B degradation, which is also controlled by APC. Rising Cdk1 activity promotes CDC20 to bind and activate the APC (Jin et al., 2010). Active APC^CDC20^ ubiquitinates cyclin B1 and thereby marks it for degradation (Hampl and Eppig, 1995); low MPF activity then facilitates anaphase entry. Activated APC also destroys securin, separase becomes active and hydrolyzes cohesin (importantly, centromeric cohesin holding the chromatids together is protected), and as a result homologous chromosomes can become separated (Herbert et al., 2003). During MI in oocytes, SAC activity is weak compared to its activity during mitosis, so chromosomal alignment abnormalities are often not detected (Nagaoka et al., 2011). This seems to be the reason for the high rates of incorrect bivalent segregation that leads to aneuploidy in oocytes. The cell cycle then progresses to telophase and upon completion of meiosis I a large secondary oocyte and a tiny first polar body are formed; the latter simply serves as the dumping ground for half of the chromosomes.

After the first round of meiosis a brief interphase known as interkinesis follows, during which no DNA replication takes place and no nuclear envelope forms, and the oocyte immediately begins the second round of meiosis. The cell cycle soon becomes arrested again, this time at the metaphase stage. The second meiotic arrest is sustained by high MPF activity, which blocks entry into anaphase. This is achieved by the inhibition of cyclin B1 destruction and an increase in Cdk1 activation. Cyclin B1 destruction is prevented because APC is inhibited by the early mitotic inhibitor 2 (Emi2; whose activity in turn is controlled by the cytostatic factor [CSF]), and also, because Cdk1 activity is kept at a high level via dephosphorylation by the phosphatase Cdc25A. In addition, APC inhibition also prevents the degradation of securin, so separase cannot hydrolyze cohesin and the bivalents cannot segregate (Sanders and Jones, 2018). This arrest happens shortly before ovulation, so that at ovulation mammalian eggs are released from the ovarian follicles while arrested at the MII stage. Maintaining the second meiotic arrest and preventing entry into the embryonic cell cycles without sperm makes “physiological sense” partly because term development of parthenogenetic embryos that form without the contribution of the male gamete would not be possible anyway due to the absence of crucial paternal genes, and also because this way the growth of potentially cancerous cells in the female genital tract can be prevented (Jones, 2007).

The intracellular organelles of the oocyte undergo significant reorganization during maturation. Some of the most prominent changes occur to the ER. In GV-stage mouse oocytes, the ER was found to be distributed throughout the cytoplasm with some of the ER accumulated around the germinal vesicle and as isolated ER clusters in the cytoplasm (Mehlmann et al., 1995; Kline et al., 1999; FitzHarris et al., 2003). During germinal vesicle breakdown, the ER becomes concentrated and forms a prominent ring around the germinal vesicle and then disperses throughout the cytoplasm. Finally, in mature oocytes the ER forms a fine reticular network throughout the cell with numerous dense accumulations (clusters) in the cortex. Although there are species-specific differences, this distribution of the ER in the cytoplasm serves a critical role during Ca^2+^ signaling at fertilization (Satouh and Sato, 2023). In addition, the sensitivity of the ER to Ca^2+^ release also increases during maturation. Concomitant with the ER reorganization during maturation, there is a 20%–90% increase in the immunoreactive mass of the inositol 1,4,5-trisphosphate receptors (IP_3_Rs) located in the ER membrane (Mehlmann et al., 1996; Fissore et al., 1999). In immature oocytes, the IP_3_Rs show homogeneous distribution in the entire cytoplasm except around the germinal vesicle. During maturation, the IP_3_Rs reorganize and become distributed in the cytoplasm in a more reticular manner with cortical clusters near the plasma membrane. This structural reorganization is thought to be important to acquire the ability to generate repetitive Ca^2+^ oscillations at fertilization (Mehlmann et al., 1996). The amount of Ca^2+^ stored in the ER also increases during maturation (Jones et al., 1995; Wakai et al., 2012). Due to the changes in these ER characteristics the Ca^2+^ signals that can be stimulated in the egg differ markedly depending on the maturation state of the female germ cell. Specifically, compared to Ca^2+^ signals found in eggs, those in immature oocytes have reduced amplitudes and durations, reduced sensitivity to IP_3_, lower oscillation frequencies, and the Ca^2+^ rises fail to propagate globally across the entire oocyte (reviewed by Stricker, 2006).

Mitochondria also undergo significant reorganization during maturation. These organelles provide energy in the form of ATP, which is critically important for the maturing oocyte (Stojkovic et al., 2001). During oocyte growth and the early phases of maturation, the oocyte is connected to cumulus cells that provide pyruvate to be used by the germ cell’s mitochondria to generate ATP, they can also supply the oocyte with ready-made ATP. Following ovulation however, the egg becomes uncoupled from the supporting somatic cells and must rely on its own mitochondria to generate ATP (Downs, 1995; Johnson et al., 2007). Mitochondrial activity also shows unique changes during oocyte maturation. ATP content increases significantly in pig (Brevini et al., 2005), bovine (Stojkovic et al., 2001) and mouse (Yu et al., 2010) oocytes during maturation and it is correlated with developmental success (Stojkovic et al., 2001; Van Blerkom et al., 1995). In immature oocytes, the mitochondria have a more peripheral position and at germinal vesicle breakdown they aggregate around the germinal vesicle as a ring-like structure. After the germinal vesicle breaks down the mitochondria disperse throughout the cytoplasm (Dumollard et al., 2006; Yu et al., 2010). This redistribution of mitochondria is accompanied with three distinct increases in cytosolic ATP levels in mouse oocytes that are associated with discrete events of oocyte maturation, with the third peak corresponding to polar body extrusion (Yu et al., 2010). The number of mitochondria also increases during oocyte development. Primordial germ cells soon after formation contain less than 10 mitochondria and by the time they become oogonia this number increases to 200. The number of mitochondria in primary oocytes is about 6,000, which then jumps up to more than 100,000 by the end of cytoplasmic maturation (Trimarchi et al., 2000; Cummins, 2004).

The localization dynamics of the ER follow a similar pattern as that of the mitochondria, which suggests a functional link between the two organelles. A prominent example of this inter-organelle coordination is observed in mouse oocytes. GV-stage oocytes are reported to undergo spontaneous Ca^2+^ oscillations, during which Ca^2+^ is periodically released from the ER into the cytosol (Wakai and Fissore, 2019). The released Ca^2+^ is then absorbed and stored by the mitochondria and this is believed to stimulate mitochondrial ATP production. The Golgi apparatus, a delivery center and the site for protein processing, also undergoes redistribution. The Golgi receives proteins and lipids from the ER and exports them to endosomes, lysosomes, or the plasma membrane. In GV-stage oocyte the Golgi apparatus is dispersed throughout the cytoplasm and at GVBD it undergoes drastic fragmentation (Moreno et al., 2002). Cortical granules are formed from the Golgi complex. In the GV-stage oocyte the granules are distributed in clusters throughout the cytoplasm and by the end of maturation they localize in the cortex, below the plasma membrane (Sathananthan et al., 1985). Cortical granules are secretory granules that have important roles during fertilization to prevent polyspermy (Liu, 2011). Overall, the changes during maturation, a process that begins with germinal vesicle breakdown and ends when the cell cycle is arrested in metaphase of the second meiotic division, empowers the egg to interact appropriately with the fertilizing sperm.

## 3 The signaling toolkit

Early during the course of evolution, cells of living organisms selected ionized calcium for signaling (Clapham, 2007). Signaling was essential to adapt to an ever-changing environment and for the purpose they needed messengers. Requirements for such messengers were that 1) their concentration must be able to fluctuate with time, i.e., their level in resting cells must be low but they need to be rapidly produced and released when needed, and 2) they must be capable of influencing the function of proteins. Ca^2+^ can fulfill both requirements. Cells spend a lot of energy to keep their intracellular Ca^2+^ levels approximately 20,000 times lower than the extracellular environment and thus they can generate a signal by rapidly increasing the Ca^2+^ level in the cytosol. Also, protein function depends on shape and charge, and Ca^2+^ can change these features by binding to the proteins. Because of these characteristics, Ca^2+^ became the most ubiquitous signal transduction element in living organisms involved in a wide range of biological processes. In neurons, skeletal muscle and heart muscle neurotransmitters trigger a Ca^2+^ influx to induce changes; antigen stimulation of immune cells leads to a Ca^2+^ influx that promotes an immune response against the invading pathogens; and Ca^2+^ entry through sperm-specific CatSper channels in the sperm tail induces hyperactivated motility prior to fertilization, just to name a few (reviewed by Cai et al., 2015).

Although it is essential for signaling, extended exposure to high Ca^2+^ levels is detrimental to the cell, probably because Ca^2+^ precipitates phosphate, another essential component of cellular physiology. Because of this, cells chelate, compartmentalize, or extrude Ca^2+^ (Clapham, 2007). Calcium chelators can protect cells from a toxic Ca^2+^ overload; a common chelator of Ca^2+^ in cells is the EF hand domain, which is present in a large number of proteins (Nakayama and Kretsinger, 1994). In eukaryotic cells the smooth ER is the main Ca^2+^ storage compartment, often occupying more than 10% of the cell volume (Lam and Galione, 2013). Ca^2+^ accumulates in its lumen where it can reach a concentration of more than 100 µM. In the ER, Ca^2+^ is stored attached to luminal Ca^2+^-binding proteins, these can be classified as either buffers or chaperones. Buffer proteins including calsequestrin and calreticulin simply bind Ca^2+^ with high capacity and hold it in the ER; chaperones such as calnexin assist in protein folding and quality control while they might also have a role in modulating Ca^2+^ signaling (Berridge, 2002). The ER actively sequesters Ca^2+^ into its lumen using SERCA (smooth endoplasmic reticulum Ca^2+^ ATP-ase) pumps that use ATP for the work. The SERCA pump family is encoded by three different genes (SERCA 1, SERCA 2, and SERCA 3), and from these genes seven different isoforms are expressed (SERCA 1a/1b, SERCA 2a/2b, and SERCA 3a/3b/3c) (Periasamy and Kalyanasundaram, 2007). In mouse eggs the presence of SERCA2b protein was demonstrated (Wakai et al., 2013). Finally, an increasing amount of experimental data indicate that other organelles such as mitochondria and the Golgi apparatus are also able to sequester Ca^2+^ and thus play a role in Ca^2+^ homeostasis and signaling (Bagur and Hajnóczky, 2017). The mitochondrial calcium uniporter (MCU) and secretory-pathway calcium ATPase (SPCA) can mediate Ca^2+^ uptake into these organelles (Duchen, 2000; Wu et al., 2023). Ca^2+^ uptake by the mitochondria not only assists in Ca^2+^ buffering but also promotes ATP synthesis, and this Ca^2+^-driven ATP production seems to be a major contribution of mitochondria to the Ca^2+^ homeostasis in mammalian eggs (Hajnóczky et al., 1995). By measuring intracellular and mitochondrial Ca^2+^ levels in mouse eggs simultaneously it has been found that the Ca^2+^ spikes are accompanied by parallel increases in the concentration of mitochondrial Ca^2+^, which stimulates ATP production (Dumollard et al., 2008). It has subsequently been shown that this Ca^2+^-driven ATP output is important to power the SERCA pumps during refilling of the ER (Wakai et al., 2013).

During signaling, the Ca^2+^ stored in the ER is released via two types of Ca^2+^ release channel receptors, IP_3_Rs and ryanodine receptors. In mammalian cells the IP_3_R predominates (reviewed by Berridge, 2009). The receptors have four subunits that span the ER membrane and form the channel pore. There are three IP_3_R isoforms (IP_3_R1, IP_3_R2, and IP_3_R3) with slightly different properties; mammalian eggs overwhelmingly express IP_3_R1, although they have the other isoforms as well (Parrington et al., 1998; Fissore et al., 1999). The opening of the IP_3_R requires binding by IP_3_ and Ca^2+^, low cytosolic Ca^2+^ levels stimulate the discharge of the stored Ca^2+^, whereas high levels inhibit it (Iino, 1990).

Once a Ca^2+^ rise is generated, the resting intracytoplasmic Ca^2+^ level must be restored. Some of the cytosolic Ca^2+^ is pumped back into the stores by the SERCA pumps described above, while the rest is removed from the cell through the plasma membrane. This is done primarily by the plasma membrane Ca^2+^ ATP-ase (PMCA) pumps that use ATP to pump Ca^2+^ against the immense concentration gradient to move Ca^2+^ out of the cell (reviewed by Carafoli, 1991). In addition, Na^+^/Ca^2+^ exchangers (NCX) located in the plasma membrane can move one Ca^2+^ out of the cell in exchange for three Na^+^ (Blaustein and Lederer, 1999). There is evidence for the function of both mechanisms in eggs (Carroll, 2000; Machaty et al., 2002b). In somatic cells another membrane protein, the Na^+^/Ca^2+^-K^+^ exchanger (NCKX) has also been described, this exchanger cotransports one Ca^2+^ with one K^+^ in exchange for four Na^+^ (Altimimi and Schnetkamp, 2007).

The removal of Ca^2+^ from the cell can be so effective that an influx mechanism may be needed to restore intracellular Ca^2+^ levels for proper cell function. Cells can use a number of mechanisms to accomplish this including receptor-operated channels (ROCs), voltage-gated channels (VGCs), transient receptor potential (TRP) channels, and ORAI channels (reviewed by Wakai et al., 2019). Two classes of voltage-gated calcium channels have been described. High-voltage-gated channels such as P/Q, N, R and L type channels are activated by strong depolarization. Low-voltage-gated channels in the other class require lower depolarization for opening, these are known as T-type channels (or Ca_V_3.1-Ca_V_3.3) (Arikkath and Campbell, 2003). The presence of Ca_V_3.2 was demonstrated in mouse eggs (Peres, 1986; Bernhardt et al., 2015). It was found that GV-stage oocytes show larger Ca_V_3.2-mediated currents compared to mature eggs. In addition, mice lacking Ca_V_3.2 channels were still able to display the Ca^2+^ oscillations at fertilization indicating that the function of these channels is to mediate the Ca^2+^ influx in GV-stage oocytes and prepare them to become capable of mounting the repetitive Ca^2+^ transients at fertilization. Because mouse eggs show only a slight change in the membrane potential during fertilization (Igusa et al., 1983; Jaffe and Cross, 1984) it remains a conundrum why these voltage-gated channels are expressed and functional in these cells. The presence of TRP channels have also been shown in mammalian eggs (Machaty et al., 2002a; Carvacho et al., 2013). In somatic cells such channels mediate Ca^2+^ entry in response to a great number of stimuli during the perception of pain, temperature, different kinds of taste, pressure, and vision. They are known to be modulated by protein kinase C (PKC) (Mandadi et al., 2011). In mouse eggs the expression of TRPV3 and TRPM7 was demonstrated and it was shown that the expression level changes during the course of maturation (Carvacho et al., 2013; Carvacho et al., 2016). Finally, components of the store-operated Ca^2+^ entry (SOCE) mechanism are also present in mammalian oocytes and eggs. In many non-excitable cells Ca^2+^ influx across the plasma membrane is stimulated in response to store depletion (Putney, 1990). One major SOCE component is the STIM protein, which is located in the ER membrane and serves as a sensor of Ca^2+^ content (Liou et al., 2005). Upon store depletion, STIM proteins oligomerize, translocate to plasma membrane-adjacent regions and open the other major SOCE component, the ORAI channel, which leads to Ca^2+^ influx (Feske et al., 2006). Two STIM proteins (STIM1 and STIM2) and three ORAI proteins (ORAI1, ORAI2, and ORAI3) have been identified in mammalian cells. The presence of STIM1 and ORAI1 has also been demonstrated in porcine and mouse eggs (Koh et al., 2009; Yu et al., 2009; Wang et al., 2012).

Changes in Ca^2+^ concentration inside of cells can be detected by Ca^2+^ sensor (also known as trigger or adaptor) proteins. These Ca^2+^ binding proteins have the ability to bind Ca^2+^ and undergo a significant conformational change that allows them to bind specific target proteins and modify their function. The best studied of these proteins is the ubiquitous Ca^2+^ sensor calmodulin. This protein changed little during the course of 1.5 billion years of evolution, clearly demonstrating the evolutionary importance of Ca^2+^ signaling (Clapham, 1995; Clapham, 2007). Equipped with four EF-hand Ca^2+^-binding motifs calmodulin amplifies the small size of Ca^2+^ to the scale of proteins by changing its own conformation upon Ca^2+^ binding. In fertilized eggs, calmodulin mediates the effects of Ca^2+^ by activating Ca^2+^/calmodulin-dependent protein kinase II (CaMKII). Another calmodulin target, the protein phosphatase calcineurin has also been described in T cells of the immune system. Calcineurin serves a key role in activating the transcription factor called nuclear factor of activated T cells (NFAT) by dephosphorylating it (Rusnak and Mertz, 2000). The presence and arrangement of the different components of the signaling toolkit in the egg allows for the creation and maintenance of the Ca^2+^ signal that the fertilizing sperm generates to stimulate embryo development.

## 4 The sperm protein that activates the egg

The fundamental question regarding fertilization is: how does the sperm activate the egg? That egg activation is a chemical process and it involves changes in the concentration of ions in the egg cytoplasm was first proposed by Jacques Loeb towards the end of the 19th century (Loeb, 1899). He based his idea on the observation that sea urchin eggs underwent parthenogenetic activation after being held in seawater that contained increased levels of ions. It was later established that the fertilizing sperm activates the egg’s developmental program by stimulating an increase in its cytosolic free Ca^2+^ concentration (Mazia, 1937; Ridgway et al., 1977). The major question then became one of how the sperm causes the Ca^2+^ increase in the egg. It became clear that the Ca^2+^ came from internal sources and it was also established that IP_3_ stimulated its release (Whitaker and Irvine, 1984; Busa et al., 1985; Miyazaki, 1988). Following decades of intensive research, the prevailing hypothesis was that the sperm binds to a transmembrane receptor on the surface of the egg and by doing so it stimulates the enzyme phospholipase C to generate IP_3_ (Shilling et al., 1994). However, the egg surface receptor to which the sperm can bind, and whose stimulation would lead to IP_3_ production and Ca^2+^ release have never been found. The other major line of thinking was the so-called fusion hypothesis. Experiments with mouse and sea urchin eggs revealed that following binding of the sperm to the egg plasma membrane, fusion of the two gametes took place prior to Ca^2+^ release (McCulloh and Chambers, 1992; Lawrence et al., 1997). The obvious explanation to this was that the sperm might contain a factor that diffuses into the egg cytoplasm and causes Ca^2+^ release.

Interestingly, it was also Loeb who first suggested that the sperm contains some factor that causes egg activation (Loeb, 1913). He claimed that the sperm delivers two substances to the egg, one of which (a “lysin”) acts on the surface to form a fertilization envelope, and another factor whose role is to prevent degeneration of the egg following formation of the fertilization envelope. Although many of his statements today are hard to reconcile with our current understanding of egg activation, Loeb was fascinated by the way embryo development is stimulated and made outstanding contributions to the field. The first experimental evidence that the sperm may contain such a factor came from experiments that demonstrated that the microinjection of a sperm extract into sea urchin eggs led to the formation of the fertilization envelope, an indication of egg activation (Dale et al., 1985). Later it was demonstrated that extracts from mammalian sperm could also induce activation of mouse, rabbit and hamster eggs (Swann, 1990; Stice and Robl, 1990). Importantly, the mammalian sperm extracts were also able to trigger repetitive Ca^2+^ oscillations in eggs of various species including hamster, mouse, human, bovine and pig (Swann, 1990; Swann, 1994; Homa and Swann, 1994; Wu et al., 1997; Machaty et al., 2000). The generation of repetitive oscillations is important because several stimuli can cause an increase in the cytosolic Ca^2+^ concentration but triggering a series of Ca^2+^ spikes was only possible by using spermatozoa. Another discovery that supported the sperm factor hypothesis was the successful application of intracytoplasmic sperm injection (ICSI) to treat male factor infertility (Palermo et al., 1992). As it was demonstrated later, injecting the sperm into the egg cytoplasm caused repetitive Ca^2+^ oscillations that could not be mimicked by sham injections (Tesarik et al., 1994; Nakano et al., 1997), further supporting the theory that the sperm activates the egg via a diffusible factor and not by binding to a cell surface receptor. The egg activating factor was shown to be located in the sperm head, in the perinuclear matrix (Kimura et al., 1998).

An important step in the identification of the sperm factor came when it was discovered that mammalian sperm extracts contained the enzyme phospholipase C that was active enough to generate IP_3_ to cause Ca^2+^ oscillations (Jones et al., 1998). This indicated that the sperm factor could be a PLC, although as follow-up studies determined it was unlike any of the known PLC isoforms. The factor was eventually identified as a novel PLC isoform termed PLCζ (PLCzeta), a sperm-specific protein (Saunders et al., 2002). It is present in the sperm of many different mammalian species and its ability to cause Ca^2+^ oscillations is evolutionary conserved (Cox et al., 2002; Rogers et al., 2004; Kurokawa et al., 2005; Yoon and Fissore, 2007). Its gene is present in all mammalian genomes sequenced to-date, and also in some birds and fish (Swann, 2022). PLCζ has been shown to induce Ca^2+^ oscillations in mouse, human, pig, and cow eggs (reviewed by Swann, 2023). The protein is localized in the sperm head and diffuses into the egg after sperm-egg fusion, this explains why microinjection of the sperm into the egg during intracytoplasmic sperm injection causes Ca^2+^ oscillations (Sato et al., 1999). It is also present in cytosolic sperm extracts that were shown to stimulate repetitive Ca^2+^ transients after injection into eggs (Saunders et al., 2002). The specific location of this protein is within the post-acrosomal region of the sperm, which is where gamete fusion begins. PLCζ is present in this region at a concentration that can stimulate repetitive Ca^2+^ transients in the egg (the amount of PLCζ in a mouse spermatozoon is 20–50 fg and the microinjection of 4–8 fg of the protein has been shown to be capable of inducing Ca^2+^ oscillations) (Saunders et al., 2002).

The molecular structure of PLCζ has been determined in great detail. PLCζ has a molecular weight of ∼70 kDa and is the smallest of all PLC isoforms (Thanassoulas et al., 2022). The enzyme lacks the N-terminal pleckstrin homology (PH) domain that is present in other PLC isoforms, and instead contains four N-terminal EF hand domains, X and Y catalytic domains separated by the XY-linker, and a C2 domain at the C-terminus. The EF hands contain Ca^2+^ binding residues and provide the enzyme with high Ca^2+^ sensitivity; deletion or mutation of these conserved Ca^2+^ binding residues abolish the Ca^2+^-induced activity of PLCζ. In effect, the EF hands act as molecular switches controlled by Ca^2+^ spikes. The X and Y catalytic domains are responsible for the catalysis of the membrane lipid phosphatidylinositol 4,5-bisphosphate (PIP_2_); a mutation in this region causes a loss in the enzyme’s ability to generate IP_3_ and trigger Ca^2+^ oscillations (Nomikos et al., 2011). C2 domains are known as Ca^2+^-dependent membrane-targeting modules involved in targeting proteins to phospholipid-containing cell membranes (Nalefski and Falke, 1996; Zheng et al., 2000). Interestingly, the C2 domain of PLCζ has no predicted Ca^2+^ binding site, nevertheless deletion of this domain abolishes the ability of PLCζ to induce Ca^2+^ oscillations (Nomikos et al., 2005). It was proposed that it facilitates the anchoring of the enzyme to membranes through its interaction with specific phosphoinositides (Thanassoulas et al., 2022). Finally, the XY-linker plays multiple roles in influencing PLCζ action. As mentioned above, unlike other isoforms PLCζ does not have a PH domain that usually functions to bind PIP_2_ within biological membranes. It is believed however, that positively charged residues within the XY-linker target the enzyme to PIP_2_, possibly via electrostatic interactions (Nomikos et al., 2007). This idea is supported by the finding that a decrease in the net positive charge of the XY-linker resulted in a decline in the enzyme’s ability to bind PIP_2_ or to stimulate Ca^2+^ transients after microinjection into eggs (Nomikos et al., 2011). The XY-linker also contains a predicted nuclear localization signal (NLS) sequence, which is a potential binding site for the nuclear transport receptor (NTR); this sequence may be important in the regulation of PLCζ function, at least in the mouse (Larman et al., 2004). A great number of mutations in the PLCζ gene have been described in patients with egg activation deficiencies. The mutations affected various domains of the protein (but primarily the X and Y catalytic domains) leading to fertilization failure, this highlights the importance of PLCζ in fertilization (reviewed by Thanassoulas et al., 2022).

Eggs of different species have different sensitivities to PLCζ. For example, mouse eggs are much more sensitive to human PLCζ than human eggs, the difference in sensitivity is about 30-fold (Yu et al., 2008). In fact, mouse eggs are about 10 times more sensitive to IP_3_-induced Ca^2+^ release as well compared to human eggs (Storey et al., 2021). This implies that something in mouse eggs sensitizes IP_3_Rs that is absent in human eggs. It has recently been proposed that the factor that regulates IP_3_Rs may be the concentration of ATP (Swann, 2023). Somatic cells express mostly IP_3_R2s and IP_3_R3s while mammalian eggs possess primarily IP_3_R1s. IP_3_R1s differ significantly in the way they are regulated by ATP: their open probability increases in response to ATP, whereas IP_3_R3s need about 10 times higher ATP levels to open and IP_3_R2s are not gated by ATP (Tu et al., 2005). This suggests that mammalian eggs are highly sensitive to ATP modulation. The IP_3_R1 was shown to be sensitive to the Mg^2+^-free form, or ATP^4−^. Approximately 95% of the ATP in the cytoplasm is bound to Mg^2+^ as MgATP^2−^, which is the form that supplies the energy to many biological processes (Mak et al., 1999). However, the total ATP concentration in mouse eggs was found to be ∼3.3 mM (Storey et al., 2021), so the ATP^4−^ level in such eggs seems to be high enough (∼200 µM) to modulate the opening of IP_3_R1s. In contrast, the total ATP concentration in human eggs is about 1.4 mM (which is equivalent to ∼90 µM of ATP^4−^), and hamster eggs may have even lower ATP levels (Van Blerkom et al., 2003). Thus, ATP levels seem to be different in the eggs of various mammalian species and this may explain their different sensitivity to PLCζ.

Other sperm proteins have also been proposed as possible candidate factors, these include the postacrosomal sheath WW domain-binding protein (PAWP; Wu et al., 2007), a truncated c-kit tyrosine kinase (Sette et al., 2002), and an extramitochondrial form of citrate synthase (Harada et al., 2007). These proteins have been suggested to trigger Ca^2+^ release and subsequent activation in mouse and human eggs. However, independent laboratories have been unable to verify the reported results and until then it is difficult to accept these candidate proteins as sperm proteins with a role in fertilization (Swann, 2023). Finally, there seems to exist a secondary factor in the sperm that may serve as a backup mechanism. This factor is observable only when PLCζ is absent but its exact identity remains elusive. When mouse eggs were fertilized *in vitro* with sperm lacking PLCζ, they showed 1 to 4 Ca^2+^ transients that occurred approximately 1 h after the sperm fused to their plasma membrane (Hachem et al., 2017; Nozawa et al., 2018). It is puzzling how these sperm can generate such Ca^2+^ spikes and the possibility exists that they contain a second factor which, when introduced into the egg, generates IP_3_ and causes the release of stored Ca^2+^. Interestingly, in the same studies, when PLCζ KO sperm were injected into the eggs via intracytoplasmic sperm injection, no Ca^2+^ oscillations occurred. Thus, the second factor seems to work during IVF but not during ICSI. In addition, the putative second factor’s effect is markedly slower: when the eggs were fertilized with sperm lacking PLCζ, the Ca^2+^ oscillations were initiated with a distinct delay of approximately 1 h following sperm-egg fusion. The belated Ca^2+^ transients caused delayed cortical granule exocytosis and as a result many zygotes were polyspermic. Identification of this putative second sperm factor will hopefully help in understanding these rather strange characteristics. Although questions remain that need to be answered, accumulating evidence shows that PLCζ is currently the only moiety that successfully fulfills all the requirements to be considered the predominant mammalian egg-activating factor.

## 5 Calcium signaling at fertilization

In most species, a rise in the intracellular free Ca^2+^ concentration is triggered by the fertilizing sperm and forms the stimulus for the egg-embryo transition. Without fertilization the eggs typically die within 48 h, but the fertilizing sperm triggers a series of physiological and biochemical changes that causes activation of the oocyte’s developmental program. The Ca^2+^ signal is evolutionarily conserved, which is striking considering the fact that eggs of different species arrest at different stages during meiosis (Stricker, 1999). Ca^2+^ is both necessary and sufficient for egg activation at fertilization in mammals (Kline and Kline, 1992a). PLCζ delivered into the egg by the sperm hydrolyzes PIP_2_ into IP_3_ and diacylglycerol (DAG). IP_3_ then binds to its receptor on the surface of the ER, which leads to the release of stored Ca^2+^ into the cytosol, while DAG can activate the enzyme PKC. In lower species there is a single Ca^2+^ rise that crosses the egg starting at the site of sperm fusion; this single Ca^2+^ elevation initiates embryo development. In mammalian species however, the initial Ca^2+^ rise is followed by a series of transients that last for several hours, this was first described by Cuthbertson and Cobbold (1985). The long-lasting stimulus seems to be needed to properly activate the egg. There are two major events that are triggered by Ca^2+^: cortical granule exocytosis to prevent the fusion of additional sperm cells to the egg, and the stimulation of cell cycle resumption. Results of experiments where the number of Ca^2+^ spikes generated in the eggs was tightly controlled revealed that in order to trigger complete cortical granule exocytosis and to induce exit from the MII arrest, multiple transients were needed (Lawrence et al., 1998; Ducibella et al., 2002; Ozil et al., 2005). In addition, fewer transients were needed to stimulate cortical granule release than cell cycle resumption, probably because it is physiologically more urgent to prevent the entry of additional sperm cells than it is to stimulate the completion of meiosis.

In mouse and hamster eggs it has been shown that the initial Ca^2+^ rise takes the form of a wave of Ca^2+^ release that crosses the egg; the wave starts at the point where the sperm fused to the egg and crosses the egg in about 5–10 s (Miyazaki et al., 1986; Deguchi et al., 2000). Earlier it was believed that after the first 2 to 3 transients the Ca^2+^ rises begin synchronously in the entire cytoplasm. However, it was later determined that subsequent Ca^2+^ elevations are also waves, but the waves are increasingly faster (after about 15 min the waves cross the egg in ∼1 s) and their point of origin in the cortex varies from one transient to the other (Deguchi et al., 2000). The reason for this shift in the wave initiation point is probably the diffusion of PLCζ in the egg cytoplasm. During the first Ca^2+^ wave, PLCζ is localized in the area where the sperm fused with the egg plasma membrane and this is the point from which the wave originates. The molecular weight of PLCζ is ∼70 kDa and proteins of this size require about 10 min to cross the mouse egg by diffusion (Swann, 1996). After ∼10 min PLCζ will have diffused throughout the entire cytoplasm, which explains why the wave initiation point varies in the cortex. These subsequent waves also have a higher speed, which is attributable to the unique way PIP_2_ is distributed in the egg cytoplasm. As mentioned above, PLCζ generates the Ca^2+^-releasing second messenger IP_3_ by hydrolyzing PIP_2_. PIP_2_ is typically localized in the plasma membrane of cells but interestingly, in mouse eggs, most PIP_2_ is found to be present in small vesicles that are distributed throughout the entire cytoplasm (Yu et al., 2012; Sanders et al., 2018). It is not entirely clear why PIP_2_ is present in these cytoplasmic vesicles but it may be related to the mechanism by which Ca^2+^ oscillations are maintained in eggs. The initial Ca^2+^ spikes at fertilization can be described by so-called IP_3_-based models; such models involve the positive feedback of Ca^2+^ on the IP_3_R (Sneyd et al., 2006; Politi et al., 2006). Mouse and human eggs have mostly IP_3_R1s (Miyazaki et al., 1992; Lee et al., 2010), which show a bell-shaped sensitivity to Ca^2+^ when stimulated by IP_3_ (Foskett et al., 2007). The IP_3_R opens in response to IP_3_ and the opening is enhanced by increasing levels of Ca^2+^. This Ca^2+^-enhanced Ca^2+^ release operates for the initial part of the Ca^2+^ transient but the IP_3_R closes when the Ca^2+^ concentration in the cytosol reaches the micromolar range. However, it has been demonstrated in mouse eggs that later sperm-induced Ca^2+^ oscillations can be explained by a different model, the Ca^2+^-induced regenerative IP_3_ formation model (Sanders et al., 2018). According to this model, PLCζ generates IP_3_ that causes Ca^2+^ release from the ER. In turn Ca^2+^ stimulates further PLCζ activity (PLCζ contains four EF hand domains that are responsible for the protein’s high sensitivity to Ca^2+^; (Nomikos et al., 2005)), this leads to more IP_3_ production and hence more Ca^2+^ release. So, the Ca^2+^ oscillations at fertilization are maintained by PLCζ through a positive feedback mechanism based upon Ca^2+^-induced IP_3_ generation (Swann, 2023). The model also explains the importance of PIP_2_ located in cytoplasmic vesicles. If PIP_2_ hydrolysis happened exclusively at the plasma membrane, the relatively slow diffusion of IP_3_ would limit the speed of the propagating Ca^2+^ wave, which, as mentioned above, reaches the speed of about 1 sec a few minutes after sperm-egg fusion. The ER is spread throughout the egg cytoplasm and is in close proximity with the PIP_2_ vesicles that are themselves about 2 µm apart from one another (Sanders et al., 2018). With this arrangement the IP_3_ generated is within a few micrometers from the IP_3_Rs, this short distance facilitates the rapid release of Ca^2+^ from the stores and the rapid propagation of the Ca^2+^ wave. The finding that in fertilized mouse eggs there is no detectable PIP_2_ hydrolysis in the plasma membrane also supports the notion that PLCζ generates IP_3_ from cytoplasmic vesicle-resident PIP_2_ (Yu et al., 2012).

The level of IP_3_ also oscillates in phase with Ca^2+^ following gamete fusion. When changes in the IP_3_ concentration was monitored in mouse eggs using the IP_3_ indicator IRIS-2.3TMR, it was found that there was a small monotonic IP_3_ increase during the first few Ca^2+^ spikes, followed by distinct oscillations in IP_3_ after approximately 20 min into the series of Ca^2+^ transients (Matsu-Ura et al., 2019). This shift is consistent with the diffusion characteristics of PLCζ. During the first few transients, PLCζ is localized in the area where the sperm has fused to the egg plasma membrane and the Ca^2+^ spikes will primarily be dependent on IP_3_ diffusion and IP_3_ stimulation. After about 20 min, PLCζ diffuses throughout the entire cytoplasm and the Ca^2+^ transients will be dependent on Ca^2+^-induced IP_3_ production, with IP_3_ levels oscillating in synchrony with Ca^2+^ oscillations (Swann, 2023).

As it was discussed above, Ca^2+^-stimulated PLCζ hydrolyzes PIP_2_ to generate IP_3_ and IP_3_ binds its receptor on the ER to induce the release of stored Ca^2+^. At one point during Ca^2+^ release the increase in the cytosolic Ca^2+^ level is terminated but interestingly, it is not entirely clear how. The nature of the negative feedback that decreases the intracytoplasmic Ca^2+^ concentration is somewhat vague (Swann, 2023). There are several mathematical models to describe Ca^2+^ oscillations in somatic cells; some models propose that it is the complete depletion of the ER stores that terminates Ca^2+^ release. However, in mouse eggs it was shown that complete emptying of the stores does not happen during each Ca^2+^ transient (Wakai et al., 2013; Sanders et al., 2018). Other models propose that the Ca^2+^ release is terminated by the Ca^2+^-induced desensitization of the IP_3_R (Sneyd et al., 2006; Politi et al., 2006). The fact that at high IP_3_ levels - which is expected when PLCζ is present - Ca^2+^ is not effective in closing the IP_3_R (Mak et al., 1988) argues against this theory. Thus, the exact mechanism responsible for terminating the Ca^2+^ transients remains elusive.

The sperm-induced Ca^2+^ oscillations last for several hours, in mice they stop around the time of pronucleus formation when the cell cycle enters interphase (Marangos et al., 2003; Larman et al., 2004). Evidence has shown that in fertilized mouse eggs PLCζ is sequestered into the pronuclei and this is responsible for the cessation of Ca^2+^ transients. In fact, mouse pronuclei have oocyte activating ability due to PLCζ sequestration. This was demonstrated when nuclei from 1- and 2-cell-stage fertilized mouse embryos were transferred to unfertilized eggs, the transferred (pro)nuclei caused repetitive Ca^2+^ transients, the completion of meiosis and pronuclear formation. Nuclei from parthenogenetic embryos did not trigger egg activation indicating that the ability to cause activation was specific to nuclei transferred from fertilized embryos. In addition, there are a short series of Ca^2+^ transients during the first mitosis in the mouse embryo; these are stimulated shortly after nuclear envelope breakdown and believed to be the result of PLCζ being released from the pronuclei (Kono et al., 1995). Sequence analysis of PLCζ from all species studied has shown that the protein possesses a putative nuclear localization signal in the XY-linker region (Thanassoulas et al., 2022); this sequence facilitates the accumulation of mouse PLCζ in the newly formed pronuclei. Although it is tempting to suggest that PLCζ sequestration regulates the cessation of the Ca^2+^ signal in mammalian eggs, this does not seem to be the case. The sperm-induced Ca^2+^ oscillations did not stop when the pronuclei formed in fertilized bovine eggs and continued during pronucleus migration and nuclear envelope breakdown (Nakada et al., 1995). Furthermore, following cRNA microinjection into the egg cytoplasm, human, rat, medaka fish and horse PLCζ did not translocate to the pronucleus in mouse zygotes (Ito et al., 2008; Sato et al., 2013), and bovine PLCζ failed to accumulate in the pronucleus of either mouse or bovine zygotes (Cooney et al., 2010). Thus, PLCζ sequestration into the pronuclei may serve a role in the termination of the Ca^2+^ oscillations in mouse zygotes but a different mechanism may be at play in other mammals.

Another phenomenon also seems to be important in the cessation of the Ca^2+^ oscillations. It was reported that in mouse eggs IP_3_R1s are downregulated and more than 50% of their mass is lost by the time pronuclei form (Brind et al., 2000; Jellerette et al., 2000). The degradation is due to the persistent stimulation of the phosphoinositide pathway and the continuous exposure of the receptors to IP_3_. Ultimately, the degradation of the IP_3_Rs, which appears to be mediated by the ubiquitin-proteasome pathway, is responsible for the diminished periodicity of the Ca^2+^-oscillations as the egg approaches interphase (Lee et al., 2010). By that time however, it has fulfilled its role and stimulated all the events that are associated with egg activation.

## 6 Ca^2+^ influx

An influx of Ca^2+^ across the egg plasma membrane is also required to maintain the Ca^2+^ transients. It is well documented that in Ca^2+^-free media the Ca^2+^ oscillations, either sperm-induced or triggered by intracytoplasmic injection of PLCζ, slow down or stop entirely (Igusa and Miyazaki, 1983; Wakai et al., 2013). An indication that an influx of Ca^2+^ is generated because of the release of Ca^2+^ from the ER came from experiments using thapsigargin, a SERCA pump inhibitor. Adding thapsigargin to mouse eggs blocks the SERCA pumps. Ca^2+^ slowly leaks out of the ER but the pumps are not able move Ca^2+^ back so the stores become depleted; this stimulates a Ca^2+^ entry across the plasma membrane (Kline and Kline, 1992b). It has been proposed that the Ca^2+^ influx was responsible for reloading the intracellular stores (Miyazaki, 1991). A similar phenomenon has been described in somatic cells as well. Agonist stimulation in numerous non-excitable cells stimulates Ca^2+^ release from the ER followed by an influx of Ca^2+^ across the plasma membrane (Putney, 1990; Berridge, 1990). The Ca^2+^ influx pathway is activated by store depletion and is termed store-operated Ca^2+^ entry (SOCE, previously known as capacitative Ca^2+^ entry). The Ca^2+^ influx regulated by the intracellular Ca^2+^ stores has been demonstrated in fertilized mouse eggs (McGuinness et al., 1996), while artificially induced SOCE has been shown to be present in pig and human eggs (Machaty et al., 2002a; Martín-Romero et al., 2008).

Although the mechanism of the Ca^2+^ influx in eggs is well-established and its importance during fertilization has been clearly demonstrated, the components of the pathway has remained elusive. The breakthrough in the understanding of the SOCE pathway in somatic cells came when the stromal interaction molecule 1 (STIM1) protein was identified as the sensor of ER Ca^2+^ content that links store depletion to Ca^2+^ influx (Liou et al., 2005; Roos et al., 2005). The channel component of the SOCE pathway, ORAI1, was identified soon afterwards (Feske et al., 2006; Vig et al., 2006). The presence and role of these proteins in mediating SOCE during fertilization has been demonstrated in pig eggs. When STIM1 levels are downregulated in porcine eggs prior to fertilization using siRNA, the eggs show only a single Ca^2+^ transient; additional Ca^2+^ rises cannot be detected in eggs that lack STIM1 (Lee et al., 2012). Similarly, downregulation of ORAI1 expression by siRNA microinjection also appears to block the sperm-induced Ca^2+^ oscillations (Wang et al., 2012). The Ca^2+^ entry mediated by STIM1 and ORAI1 has been found to be essential to sustain the fertilization Ca^2+^ signal in pig eggs (Wang et al., 2015), and Förster Resonance Energy Transfer (FRET) analysis has clearly indicated the repetitive interaction between STIM1 and ORAI1 during fertilization, in synchrony with the sperm-induced Ca^2+^ transients (Zhang et al., 2018). These data strongly imply that store-operated Ca^2+^ entry, mediated by an interplay between STIM1 and ORAI1 operate during fertilization in the pig.

Intriguingly, in mouse eggs the Ca^2+^ entry that maintains the sperm-induced Ca^2+^ oscillations is mediated by a different mechanism. In this species, inhibiting SOCE by specific inhibitors (Miao et al., 2012), or by the expression of protein fragments that interfere with the interaction between STIM1 and ORAI1 (Takahashi et al., 2013) does not prevent the Ca^2+^ transients at fertilization. Furthermore, in transgenic mice that lack STIM1, STIM2, or ORAI1, eggs of the knockout animals display normal Ca^2+^ influx after store depletion and importantly, show normal patterns of Ca^2+^ oscillations at fertilization (Bernhardt et al., 2017). Combined, these data indicate that as opposed to pigs, in mouse eggs SOCE is not needed during fertilization. Research then focused on TRP channels as these can serve as Ca^2+^ influx channels in several cell types (Ramsey et al., 2006), they are expressed in eggs (Petersen et al., 1995; Machaty et al., 2002a), and because certain TRP isoforms are regulated by PKC, a protein that is activated during fertilization (Hardie, 2007). An ion channel current activated by TRP agonists has been identified in mouse eggs; the current is absent in eggs of transgenic animals lacking transient receptor potential vanilloid member 3 (TRPV3) channels. However, eggs of TRPV3-knockout animals display normal Ca^2+^ oscillations upon fertilization (Carvacho et al., 2013) revealing that these channels are not essential for normal fertilization. The T-type channel Ca_V_3.2 has also been suggested to be a part of the Ca^2+^ entry mechanism. Mouse eggs lacking the α1 subunit of this channel show impaired Ca^2+^ oscillations at fertilization (Bernhardt et al., 2015) indicating their involvement in Ca^2+^ homeostasis and signaling. However, mice having non-functional Ca_V_3.2 channels are still fertile (although they show impaired fertility), which implies that additional influx mechanisms must have a role in maintaining the Ca^2+^ oscillations at fertilization. More promising results have been obtained when TRPV3 was knocked out together with Ca_V_3.2; deletion of these channels has disrupted Ca^2+^ store refilling and has a negative effect on the sperm-induced Ca^2+^ transients (Mehregan et al., 2021). The Transient Receptor Potential Melastanin 7 (TRPM7)-like channel has also been identified as a potential candidate. Such channels are present in mouse eggs and their inhibition prevents spontaneous Ca^2+^ influxes in immature oocytes, while it also disrupts the repetitive sperm-induced Ca^2+^ signal (Carvacho et al., 2016; Bernhardt et al., 2017). Finally, mouse eggs lacking both TRPM7 and Ca_V_3.2 are unable to maintain the long-lasting Ca^2+^ oscillations at fertilization and it has been concluded that together they almost completely account for the majority of Ca^2+^ influx following store depletion (Bernhardt et al., 2018). In addition, TRPM7 may also have a role in mediating Mg^2+^ homeostasis as it has been demonstrated that fertilization is more effective (Herrick et al., 2015) and the sperm-induced Ca^2+^ signal is more efficiently generated in the presence of low Mg^2+^ levels (Zhang and Machaty, 2017; Ozil et al., 2017).

Although the underlying mechanisms are different, a Ca^2+^ influx is essential in both mouse and pig eggs to maintain the Ca^2+^ oscillations at fertilization. The generally accepted reasoning is that the Ca^2+^ influx is needed to refill the intracellular stores, which in turn is important for the next Ca^2+^ transient to take place. Lately however, in light of the Ca^2+^ sensitivity of PLCζ another explanation has been offered. It has been suggested that the rapid cessation of the Ca^2+^ transients in the absence of extracellular Ca^2+^ may not be due to low Ca^2+^ levels in the ER but due to reduced levels of Ca^2+^ in the cytosol (Swann, 2023). Too low cytosolic Ca^2+^ levels may be insufficient to stimulate PLCζ to generate IP_3_. This postulation is supported by the results of an experiment using thapsigargin, the SERCA pump inhibitor. As mentioned above, incubating eggs in the presence of thapsigargin leads to the slow leak of stored Ca^2+^ from the ER into the cytoplasm. In the experiment, PLCζ was injected into mouse eggs to generate a series of Ca^2+^ transients in their cytoplasm. When the eggs were subsequently placed in Ca^2+^-free medium the oscillations stopped as expected. However, when thapsigargin was added to the holding medium the oscillations resumed and continued for more than an hour (Sanders et al., 2018). This indicates that mouse eggs can undergo repetitive Ca^2+^ transients in Ca^2+^-free medium if the cytosolic Ca^2+^ concentration is raised; the Ca^2+^ in the cytosol can stimulate PLCζ to generate IP_3_ and additional Ca^2+^ spikes are generated. This alternative explanation regarding the effect of the Ca^2+^ influx also highlights its importance in maintaining the Ca^2+^ oscillations during fertilization.

## 7 Cortical granule exocytosis

Ca^2+^ stimulates a major event of egg activation, cortical granule exocytosis, the release of the content of the cortical granules into the perivitelline space to prevent polyspermy. The process seems to be induced by a signal transduction cascade that most probably involves myosin light chain kinase (MLCK). MLCKs are Ca^2+^/calmodulin-dependent protein kinases. Following its release from the ER, Ca^2+^ binds the intracellular Ca^2+^-binding protein calmodulin, and the complex can activate a number of downstream protein kinases including MLCK. Once activated, MLCK phosphorylates (i.e., activates) the light chain of myosin and activated myosin will be able to interact with actin (Simerly et al., 1998; Kamm and Stull, 2001). MLCK and myosin are involved in the regulation of cytoskeletal-mediated events in the egg cortex indicated by the fact that ML-7, an MLCK inhibitor is able to block chromatin-induced cortical actin localization and lateral cortical granule movements in mouse eggs (Deng et al., 2005). It was also found that blocking MLCK with ML-7, or using blebbistatin, a myosin II isoform-specific inhibitor, did not decrease the activity of CaMKII and had no effect on the elevation of intracellular Ca^2+^ levels, but markedly reduced cortical granule exocytosis (Matson et al., 2006).

It has been suggested that exocytosis of the cortical granule contents alters the properties of the zona pellucida, thus prevents the entry of excess sperm into the perivitelline space. The enzyme responsible for transforming the zona pellucida is believed to be astacin-like metalloendopeptidase (Astl, also known as ovastacin; Burkart et al., 2012). Astl partially degrades the ZP2 protein, a component of the zona pellucida and thereby blocks the passage of additional sperm cells. During mouse *in vitro* fertilization, cortical reaction and ZP2 cleavage (zona hardening) reached a plateau about 40 min after the sperm-egg fusion, once the first 2 or 3 Ca^2+^ transients are completed (Satouh et al., 2017). In eggs of Astl-deficient mice the sperm cells accumulated in the perivitelline space indicating a lack of zona hardening (although curiously, no polyspermy was observed in *in vivo* fertilized eggs). During the course of this so-called zona reaction another zona pellucida glycoprotein, ZP3 is also modified and it has been found that as a result it loses its sperm receptor activity and the ability to induce the acrosome reaction (Wassarman, 1988). These changes in the structure of the zona pellucida prevent additional sperm from reaching the egg plasma membrane and ultimately guarantee normal (i.e., diploid) chromosome number in the embryo.

Interestingly, the release of the content of granules of a different type was also reported to play a role at fertilization. These granules are also located beneath the plasma membrane and have high Zn^2+^ content. As it was revealed through a series of experiments the Zn^2+^ stored in the granules is released into the extracellular milieu during the Ca^2+^ oscillations (Kim et al., 2011). Artificial depletion of the Zn^2+^ stores by treating the eggs with a Zn^2+^ chelator caused activation of mouse and porcine eggs in the absence of Ca^2+^ spikes indicating the importance of intracellular Zn^2+^ in the second meiotic arrest of the eggs (Suzuki et al., 2010; Uh et al., 2019). These so-called zinc fluxes seem to regulate meiosis through the APC inhibitor Emi2, a zinc-binding protein; zinc chelators strip Zn^2+^ from Emi2, thus causing meiotic resumption (Bernhardt et al., 2012).

## 8 Completion of meiosis

Another major event triggered by Ca^2+^ is the resumption and eventual completion of meiosis. This process is mediated via a signal transduction mechanism that is different from the one that stimulates cortical granule exocytosis. As mentioned above, Ca^2+^ released from the ER binds calmodulin, which in this case activates the Ca^2+^ sensing enzyme called calmodulin-dependent protein kinase II (CaMKII). The activity of CAMKII is oscillatory and parallels the transient elevations of Ca^2+^ in fertilized eggs (Markoulaki et al., 2003; Markoulaki et al., 2004). CaMKII in turn activates Wee1b, a protein tyrosine kinase, which inhibits Cdk1 by phosphorylation (Oh et al., 2011). CamKII also phosphorylates and thus inhibits the APC inhibitor Emi2 through Polo-like kinase 1 (Plk1; Solc et al., 2015) and as Emi2 is degraded, APC is activated (Madgwick et al., 2006). This enables the destruction of cyclin B1 as well as securin, thus stimulating cell cycle progression (Madgwick and Jones, 2007). The inhibition of Cdk1 and destruction of cyclin B1 means MPF inactivation. Interestingly, according to a recent study, fertilization causes MPF inactivation through the inhibition of Cdk1 activity without concomitant cyclin B1 degradation in bovine eggs, so species-specific differences may exist (Valencia et al., 2021). At the same time the decrease in securin results in the activation of separase, which in turn hydrolyzes centromeric cohesin that leads to the separation of sister chromatids at anaphase (Fulka et al., 1994). As it involves the segregation of sister chromatids, this second cell division is an equational division, similar to mitosis (Sanders and Jones, 2018). It is also asymmetric (just like the first round of meiosis was), leading to the formation of a one-cell embryo and a second polar body. Full completion of meiosis II requires the reformation of the nuclear envelope and chromosome decondensation, which means that another signaling component, MAPK also needs to be destroyed. MAPK levels are high during the MII arrest and high levels of MAPK activity were shown to be incompatible with the presence of a pronuclear envelope (Moos et al., 1996). The repetitive Ca^2+^ oscillations cause the destruction of MAPK and thus meiosis II is allowed to be completed and the pronuclei are formed (Gonzalez-Garcia et al., 2014).

A single rise of cytosolic Ca^2+^ is believed to be a poor activator of mammalian eggs, probably because in such eggs Emi2 is only transiently degraded. These eggs may complete their second meiotic division and extrude a second polar body but tend to re-arrest at a new metaphase without forming a pronucleus (Kubiak, 1989). When the activity of Emi2 is regained it will inhibit the APC, and as a result cyclin B1 is reaccumulated that leads to active MPF and metaphase arrest (Jones, 2007). It was suggested that in aged eggs, those that have passed the window of their normal time of fertilization, the situation is different (Fulton and Whittingham, 1978). Such eggs might be less able to maintain high MPF levels and in their case a single monotonic Ca^2+^ rise can cause egg activation effectively. Therefore, under physiological conditions the importance of the oscillatory Ca^2+^ signal may be to provide a signal that is long enough to guarantee that the egg exits meiosis (Jones, 2005). Another function of the repetitive signal seems to be its effect on long-term embryo development. When the eggs were exposed to a series of Ca^2+^ rises of varying amplitude and duration in a specifically designed activation chamber, they all underwent high rates of activation (Ducibella et al., 2006). However, their longer-term (i.e., post-implantation) development was markedly affected by the Ca^2+^ spiking regiment, with more transients having a positive effect on chromatin remodeling and reprogramming of gene expression. It is hypothesized that the repetitive Ca^2+^ spikes are important not only to stimulate the degradation of proteins whose function is to maintain the metaphase II arrest, but they may also be critical to switch on the expression of proteins whose function is needed for proper embryo development (Jones, 2007).

## 9 Conclusion

A lot has been discovered about the making of an embryo in the previous decades. The egg forms during oogenesis and by the time it reaches the site of fertilization, it acquires all the components and features that allow it to appropriately respond to the fertilizing sperm. It must be activated to become an embryo and it is clear that activation is mediated by a signaling mechanism that involves Ca^2+^. This important second messenger is released from the intracellular stores of the egg and once in the cytoplasm it interacts with downstream components of the signaling cascade that results in the exclusion of additional sperm cells to prevent polyspermy, the completion of the meiotic division and the initiation of the first mitotic cell cycle of the newly formed embryo. PLCζ emerged as the moiety that the sperm uses to generate the Ca^2+^ signal. Although it is of critical importance in the process of mammalian fertilization, there are still a number of things that need to be learned about PLCζ. For example, the putative binding partners of PLCζ within the sperm remain to be elucidated; identification of those molecules is a prerequisite to completely understand the function of this important egg-activating molecule. The most promising candidate for this role seems to be calmodulin (Leclerc et al., 2020). Calmodulin is the primary sensor of intracellular Ca^2+^ levels and it is able to interact with numerous proteins including several phospholipase isoforms. It is localized in similar regions of the sperm as PLCζ and the XY-linker region of PLCζ contains an amino acid sequence that is recognized by calmodulin (Thanassoulas et al., 2022). Future studies will determine the importance of such a PLCζ-calmodulin complex in the regulation of PLCζ function. In addition, the precise regulation of PLCζ activity in the egg cytoplasm is also unknown. PLCζ stimulates repetitive Ca^2+^ transients only in eggs and not in somatic cells (Phillips et al., 2011) and it is unclear how this is achieved. Mammalian PLCs are typically activated by interaction with other proteins, either in the cell where they are produced or in those to which they are transported (Suh et al., 2008). It is possible that a unique “egg factor” exists that interacts with PLCζ in the egg cytoplasm and enables it to induce multiple Ca^2+^ oscillations but the identity of such a factor has not been defined yet. Another distinctive feature of PLCζ is that it targets PIP_2_ in intracellular vesicles and not in the plasma membrane (Yu et al., 2012). This seems to be important to facilitate the propagation of the Ca^2+^ wave throughout the cytoplasm but the way it is regulated is currently unclear. It is possible that PLCζ identifies these vesicles by interacting with the yet-to-be identified egg factor, which may be a cytosolic binding protein or a membrane receptor (Swann and Lai, 2013). Finally, the role of ATP in the signaling process at fertilization is not completely understood although it may profoundly affect the ability of the egg to generate the signal that turns the egg into an embryo.
